# Age-Related Dietary Habits and Blood Biochemical Parameters in Patients with and without Steatosis—MICOL Cohort

**DOI:** 10.3390/nu15184058

**Published:** 2023-09-19

**Authors:** Rossella Donghia, Pasqua Letizia Pesole, Antonino Castellaneta, Sergio Coletta, Francesco Squeo, Caterina Bonfiglio, Giovanni De Pergola, Roberta Rinaldi, Sara De Nucci, Gianluigi Giannelli, Alfredo Di Leo, Rossella Tatoli

**Affiliations:** 1 National Institute of Gastroenterology—IRCCS “Saverio de Bellis”, 70013 Castellana Grotte, Italy; letizia.pesole@irccsdebellis.it (P.L.P.); sergio.coletta@irccsdebellis.it (S.C.); catia.bonfiglio@irccsdebellis.it (C.B.); giovanni.depergola@irccsdebellis.it (G.D.P.); roberta.rinaldi@irccsdebellis.it (R.R.); sara.denucci@irccsdebellis.it (S.D.N.); gianluigi.giannelli@irccsdebellis.it (G.G.); rossella.tatoli@irccsdebellis.it (R.T.); 2 Gastroenterology and Digestive Endoscopy, University Hospital, 70124 Bari, Italy; antocastellaneta@hotmail.com (A.C.); francesco.squeo@uniba.it (F.S.); alfredo.dileo@uniba.it (A.D.L.)

**Keywords:** steatosis, food intake, machine learning

## Abstract

Background: Steatosis is now the most common liver disease in the world, present in approximately 25% of the global population. The aim of this study was to study the association between food intake and liver disease and evaluate the differences in blood parameters in age classes and steatosic condition. Methods: The present study included 1483 participants assessed in the fourth recall of the MICOL study. Patients were subdivided by age (</>65 years) and administered a validated food frequency questionnaire (FFQ) with 28 food groups. Results: The prevalence of steatosis was 55.92% in the adult group and 55.88% in the elderly group. Overall, the results indicated many statistically significant blood parameters and dietary habits. Analysis of food choices with a machine learning algorithm revealed that in the adult group, olive oil, grains, processed meat, and sweets were associated with steatosis, while the elderly group preferred red meat, dairy, seafood, and fruiting vegetables. Furthermore, the latter ate less as compared with the adult group. Conclusions: Many differences were found between the two age groups, both in blood parameters and food intake. The random forest also revealed different foods predicted steatosis in the two groups. Future analysis will be useful to understand the molecular basis of these differences and how different food intake causes steatosis in people of different ages.

## 1. Introduction

The demographics of the Italian population, with 60.8 million inhabitants and the largest share of the elderly aged ≥65 years, is changing [1]. 

Aging is accompanied by progressive physiological alterations, disturbances of homeostasis, functional decline, and frailty. The elderly are more susceptible to various diseases, such as cancer, cardiovascular disease, hypertension, diabetes, chronic obstructive pulmonary disease, cerebrovascular disease, hearing loss, dementia, arthritis, and many others [2,3]. 

The liver has an optimal capacity for self-regeneration. In the healthy liver, regeneration and repair are driven by mitogenic growth factors and cytokines and by complex molecular mechanisms. However, age affects the physiological turnover and regenerative capacity of the organ [4]. Impaired autophagy is also relevant in the aging liver, as autophagy is required for the turnover of proteins and misfolded organelles, such as mitochondria under both homeostatic and pathological conditions, and for the mobilization of lipid stores during fasting [5,6].

Steatosis is now the most common liver disease in the world and is present in approximately 25% of the world’s population [7]. It can be considered a serious health problem in western and developed countries, affecting not only 30% of adults but also children and adolescents. It is the leading cause of chronic liver disease in Europe [8]. 

Steatosis is characterized by an abnormal accumulation of fat in more than 5% of hepatocytes in the absence of other causes, such as alcohol consumption (≥30 g/day for men and ≥20 g/day for women), viral hepatitis, or drugs. 

The overlap of some risk factors for the liver may favor the progression to non-alcoholic steatohepatitis (NASH), in which steatosis is associated with a state of necroinflammation [9]. 

Despite the now well-recognized role of genetic factors in the development of steatosis [10], environmental factors, such as diet and lifestyle, and chronic non-communicable diseases, including diabetes and obesity, are the main risk factors for steatosis [11]. In fact, generally, the prevalence of liver disease is higher among obese and diabetic subjects than in non-obese and non-diabetic subjects, affecting not only over 90% of obese and 60% of diabetic subjects but also over 20% of subjects of normal weight [12].

Aging is another factor that increases the incidence of the disease. Some studies have evaluated the link between physiological cellular senescence and hepatic fat accumulation, hypothesizing a role of cellular aging in the development of steatosis [13]. 

Normal aging is associated with a redistribution of body fat in both sexes, with a decrease in subcutaneous adipose tissue, an increase in visceral adipose tissue, and an accumulation of fat in ectopic sites, including the liver [14]. In fact, ectopic fat accumulation can be considered a hallmark of aging. A progressive increase in fat mass has been described as beginning around the age of 65 in men and subsequently in women [15].

Age-related dysregulation of lipid metabolism and accumulation of triglycerides in the liver contribute to organ dysfunction [14]. 

Under these conditions, an unhealthy diet can promote the development of steatosis. Biological, psychological, and socio-economic aspects, such as loss of appetite and alterations of smell and taste, can lead to a change in the composition of the diet and eating habits of the elderly [16].

Starting from the crucial role of dietary habits in health and disease prevention [17], the aim of this study is to evaluate how age affects dietary habits in a cohort of patients from southern Italy, identifying the most predictive foods for the development of steatosis using a machine learning approach.

## 2. Materials and Methods

### 2.1. Study Population

Subjects in the present study were recruited for the first time from the electoral register of Castellana Grotte, a town in southern Italy, to take part in a multicenter Italian study on cholelithiasis (MICOL). Methodological details of this population-based study have been previously published [18,19]. For this study (called MICOL IV), recall of MICOL III patients was adopted [20].

All participants signed informed consent before examination and the study was approved in line with the ethical standards of the institutional research committee of the National Institute of Gastroenterology and Research Hospital “S. de Bellis” in Castellana Grotte, Italy (DDG-CE 782/2013. The date of approval was Prot. n.144/C.E. of 15/04/2019). The study was conducted in accordance with the Helsinki Declaration of 1975. The present study adhered to the “Standards for Reporting Diagnostic Accuracy Studies” (STARD) guidelines and the manuscript was organized according to the “Strengthening the Reporting of Observational Studies in Epidemiology-Nutritional Epidemiology” (STROBE-nut) guidelines [21].

Participants were interviewed for medical history and a fasting venous blood sample was taken. The serum was separated into several aliquots. An aliquot was immediately stored at −80 °C. The second aliquot was used to test serum biochemical markers by standard laboratory techniques in our laboratory. 

Subjects were subdivided into two categories: adult versus elderly if aged ≥65 years [5]. The metabolic syndrome variable (MeS) was built based on International Diabetes Federation (IDF) criteria [22], and liver steatosis was established by abdominal ultrasound screening and graded based on liver echogenicity [23].

### 2.2. Dietary Assessments

To evaluate dietary habits, a validated food frequency questionnaire was administered during the visit, and each food (86 validated foods) was converted to mean daily intake in grams; the total was summarized in 28 food groups [20] established according to similarity type [24].

### 2.3. Statistical Analysis

Patients’ characteristics are reported as mean and standard deviation (M ± SD) for continuous variables, and as frequency and percentages (%) for categorical variables. To test the association between the independent groups (adults vs. elderly), a chi-square or Fisher test was used for categorical variables, where necessary, while the Wilcoxon Rank Mann–Whitney was used for continuous variables.

To select the predictors of the steatosis variable, a random forest (RF) was applied. RF was computed by an ensemble of binary decision trees, which could be used to select the most important variables linked to the outcomes. Variable predictiveness could be assessed using variable importance measures for both single and grouped variables [25]. Variables are considered “more important” if the variable is more frequently used for the first splits across all decision trees grown in the random forest. The parameter used for ranking was the importance score variable, calculated by adding up the improvement in the objective function given by the splitting criterion over all the internal nodes of a tree and across all trees in the forest (separately for each predictor variable). Variables with high importance were the drivers of the outcome, and their score values had a significant impact on the outcome [16]. We used another statistical methodology to evaluate the variables’ importance; in fact, predictor importance was estimated based on the minimal depth of the maximal subtree. The “Depth” was the level of the node in the tree, starting the numbering at 0 for the root node, while “Minimal depth” was the minimal depth value for the first instance of a given splitting variable. “Mean minimal depth” was the minimal depth for a variable averaged across all trees in the forest. If a predictor was influential in a prediction, then the variable was likely to occur nearer to the root rather than the leaf nodes [26]. A lower mean minimal depth of a feature represented a higher number of patients categorized in a specific group based on that feature. 

Depth is indicated by a vertical bar with the mean value. The smaller the mean minimal depth, the more important the variable and the higher up the y-axis the variable will be. The color gradient reveals the min and max minimal depth for each variable. The range of the x-axis is from zero to the maximum number of trees for the feature. We randomly split the data into training and testing subgroups to predict visual outcomes separately. 

For the adult subcohort, the training data included 75% of the sample (*n* = 544), while the remaining data (the test data) accounted for 25% (*n* = 182) and were used to test the model and minimize the heterogeneity of the obtained subsamples. In the same way, for the elderly subcohort, the training data included 75% (*n* = 568), and the remaining data accounted for 25%, (*n* = 189).

To test the null hypothesis of non-association, the two-tailed probability level was set at 0.05. The analyses were conducted with StataCorp. 2023. Stata Statistical Software: Release 18. College Station, TX, USA: StataCorp LLC., while RStudio (“Prairie Trillium” Release) was used for the plots.

## 3. Results

Males had a higher prevalence of steatosis in both the adult (66.26%) and elderly (58.87%) groups, but there was a statistically significant difference between steatosic and non-steatosic patients only in the first group (*p* < 0.001) (Table 1).

With regard to age, this had an opposite behavior between the two age categories, i.e., patients had a higher average age in the group of steatotic adults (55.63 ± 6.32 vs. 53.48 ± 6.20, *p* < 0.0001) and a lower age in the elderly (72.79 ± 5.93 vs. 74.55 ± 6.51, *p* = 0.0002). The level of education was found to be associated with the condition of steatosis in adults (*p* = 0.01), but also among the groups stratified by disease (*p* < 0.001). Fewer elderly people were ill and smokers (7.25% vs. 12.20%, *p* = 0.02), while significant differences were found between the two age groups for smoking habits, as fewer elderly people (both healthy and ill) smoked compared to subjects < 65 years of age (12.20% vs. 18.34%, *p* = 0.03, and 7.25% vs. 18.80%, *p* < 0.001). BMI, diabetes, hypertension, and MetS were more prevalent not only among disease categories in the subcohorts but also among the steatosis and non-steatosis groups (with all *p* < 0.05). Glucose levels were higher in patients with steatosis, both adults and the elderly. Furthermore, the elderly had higher levels (both non-steatotic and steatotic) than adults (*p* < 0.0001). Cholesterol did not differ in non-steatosis or steatosis conditions, but there was a significant difference (*p* < 0.0001) between the adult and elderly groups, showing higher cholesterol levels in the latter. High-density lipoprotein (HDL) was lower in steatotic adults (46.48 ± 12.18 vs. 54.62 ± 13.21, *p* < 0.0001), but this lower mean was also seen when comparing the elderly non-steatotic with healthy adults (51.60 ± 13.60 vs. 54.62 ± 13.21, *p* = 0.002). Triglycerides and insulin were higher in the steatotic subjects in both groups, with all *p* < 0.0001. The same trend was observed for homeostasis model assessment-estimated insulin resistance (HOMA-IR) (*p* < 0.0001) and red blood cells (RBC) (*p* = 0.02), with higher values in the subgroup of steatosis patients and with statistically significant differences. Furthermore, for the latter parameter, the differences were also found between the groups in the individual age groups, with lower values in the elderly than in adults (both *p* < 0.0001). Hemoglobin level was statistically significantly higher in patients with steatosis in both groups (*p* < 0.0001 and *p* = 0.03) but, comparing separately the steatotic and non-steatotic in the elderly and adult groups, elderly patients had lower levels. Only in the adult category, the hematocrit (he-MAT-uh-krit) (HCT) presented a statistically higher concentration (43.27 ± 3.37 vs. 42.10 ± 3.37, *p* < 0.0001) and mean corpuscular volume (MCV) levels were lower (85.66 ± 5.31 vs. 86.01 ± 6.97, *p* = 0.01). Mean corpuscular hemoglobin concentration (MCHC) was higher in the steatotic adults (34.03 ± 1.20 vs. 33.60 ± 1.13, *p* < 0.0001), while the level of white blood cells (WBC) was higher in patients with steatosis both in adults and in the elderly (6.33 ± 1.94 vs. 5.82 ± 2.53, *p* < 0.0001, and 6.13 ± 1.77 vs. 5.84 ± 1.61, *p* = 0.01). The condition of steatosis was also characterized by high levels of neutrophils (*p* < 0.0001), lymphocytes (*p* < 0.0001), monocytes (*p* < 0.0001), hemoglobin A1c (HbA1c) (*p* < 0.0001), aspartate aminotransferase (GOT) (*p* < 0.0001), serum glutamic pyruvic transaminase (SGPT) (*p* < 0.0001), and gamma-glutamyl transferase (GGT) (*p* < 0.0001 and *p* = 0.0003) with statistically significant values. Urea and creatinine showed higher concentrations in the steatotic category only in adults (39.17 ± 9.00 vs. 37.13 ± 9.52, *p* = 0.03, and 0.81 ± 0.17 vs. 0.77 ± 0.41, *p* < 0.0001, respectively), but in general (both steatotic and non-steatotic), the levels in the elderly compared to adults were statistically higher (42.90 ± 12.68 vs. 37.13 ± 9.52, *p* < 0.0001, and 42.94 ± 24.07 vs. 39.17 ± 9.00, *p* = 0.001, respectively). The estimated glomerular filtration rate (eGFR) (84.55 ± 9.54 vs. 86.16 ± 9.28, *p* = 0.04), and folate (7.34 ± 3.68 vs. 7.84 ± 3.74, *p* = 0.02) had lower levels in adult and also between age groups. Levels of vitamin B12 (*p* = 0.006), free triiodothyronine (FT3) (*p* = 0.001), and C-reactive protein (CRP) (*p* < 0.0001) were higher in steatotic adults with statistically significant differences. Furthermore, the latter also had higher levels in the elderly group (0.37 ± 0.73 vs. 0.12 ± 0.05, *p* = 0.05).

Table 2 analyzes the daily consumption of different foods, in the subcohorts of adults and the elderly and between steatosis and non-steatosis. Except for the consumption of potatoes, olive oil, and wine, the older patients ate less than the adult group, while significantly more red and processed meat and seafood/shellfish were consumed by the steatosis group in both age groups (*p* < 0.05). Furthermore, kilocalorie (Kcal) intake suggested higher caloric intake in older patients (2118.41 ± 858.95 vs. 1954.00 ± 747.95, *p* = 0.04).

**Figure 1:**
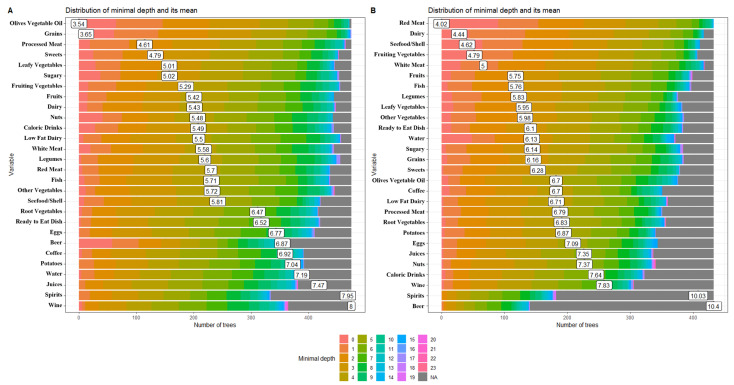
The distribution of minimal depth among the trees of the forest for the significant variables in the adult (**A**) and elderly (**B**) patient groups.

The distribution of minimal depth among the decision trees of the forests for the first four top significant variables is shown in Figure 1. These were olive and vegetable oils (3.54), grains (3.65), processed meat (4.61), and sweets (4.79) in the adult group (Figure 1A), versus red meat (4.02), dairy (4.44), seafood/shellfish (4.62), and fruiting vegetables (4.79) in the elderly sub-cohort (Figure 1B).

Furthermore, in Supplementary Table S1, we investigated the different distributions of food consumption between genders in different steatosis and age classes. In the group of non-steatotic adults, eggs (11.64 ± 10.91 vs. 9.27 ± 7.97, *p* = 0.03), leafy and fruiting vegetables (69.87 ± 77.49 vs. 48.10 ± 50.98 and 106.08 ± 104.60 vs. 75.49 ± 69.33, *p* = 0.007, respectively), root vegetable (22.46 ± 38.24 vs. 16.29 ± 36.50, *p* = 0.003), other vegetables (99.79 ± 109.74 vs. 73.00 ± 82.11, *p* = 0.04), fruits (483.07 ± 504.04 vs. 354.66 ± 412.29, *p* = 0.02), and nuts (6.57 ± 10.37 vs. 3.37 ± 4.41, *p* = 0.03) were consumed mostly by females. On the contrary, men consumed not only more red meat (27.56 ± 32.36 vs. 36.41 ± 30.18, *p* < 0.0001), processed meat (5.65 ± 9.20 vs. 6.59 ± 6.71, *p* = 0.01), grains (97.42 ± 90.94 vs. 156.90 ± 123.38, *p* < 0.0001), juices (11.80 ± 35.43 vs. 12.50 ± 26.74, *p* = 0.01), caloric drinks (7.03 ± 19.77 vs. 16.33 ± 47.66, *p* = 0.001), coffee (56.02 ± 41.15 vs. 65.03 ± 40.39, *p* = 0.03), but also alcoholic drinks, such as wine (51.99 ± 111.39 vs. 99.95 ± 136.63, *p* = 0.0001), beer (10.50 ± 45.35 vs. 42.08 ± 87.15, *p* = 0.004), and spirits (0.58 ± 1.58 vs. 2.70 ± 6.36, *p* < 0.0001).

In the steatotic group, eating behaviors were partly similar, with a higher intake in the female group of low-fat dairy (91.59 ± 119.39 vs. 69.84 ± 100.47, *p* = 0.006), white meat (30.32 ± 30.13 vs. 24.46 ± 27.00, *p* = 0.01), leafy vegetables (66.28 ± 87.94 vs. 41.46 ± 46.56, *p* = 0.004), fruiting vegetables (106.73 ± 104.76 vs. 71.66 ± 73.83, *p* = 0.0004), and other vegetables (98.25 ± 102.12 vs. 62.70 ± 77.54, *p* = 0.001). Furthermore, women had lower intake of potatoes (9.47 ± 8.90 vs. 12.75 ± 13.18, *p* = 0.006), grains (100.73 ± 91.21 vs. 147.51 ± 138.72, *p* = 0.008), and drinks, such as caloric (9.49 ± 20.72 vs. 17.61 ± 63.17, *p* = 0.03), wine and beer (42.88 ± 66.95 vs. 144.42 ± 184.04 and 17.41 ± 53.42 vs. 64.05 ± 128.50, *p* < 0.0001, respectively), and spirits (0.87 ± 2.30 vs. 2.70 ± 6.67, *p* = 0.0002), and a lower total kcal (1897.26 ± 784.01 vs. 2150.47 ± 747.30, *p* = 0.0002). 

The elderly group showed different behaviors between groups. Seafood/shellfish (2.34 ± 5.34 vs. 2.92 ± 5.24, *p* = 0.05), wine (79.82 ± 69 vs. 147.07 ± 136.14, *p* < 0.0001), and kcal intake were lower in the healthy female group. The same trend was observed in the other group with steatosis. Eggs (7.62 ± 6.35 vs. 8.70 ± 6.47, *p* = 0.03), alcoholic drinks, such as wine (81.19 ± 92.00 vs. 161.20 ± 161.22, *p* < 0.0001), beer (11.79 ± 26.00 vs. 33.60 ± 84.26, *p* = 0.002), and spirits (1.02 ± 2.03 vs. 1.97 ± 4.84, *p* = 0.002), and total kcal (2008.81 ± 992.14 vs. 2202.18 ± 733.59, *p* = 0.0007) were lower in the female group.

## 4. Discussion

The aim of this study is to examine a cohort of patients in southern Italy with and without steatosis in different age classes, and, in particular, to explore blood differences, but above all, to evaluate the association between eating habits and the development of the disease.

In the present study, higher levels of glucose, triglycerides, insulin, and other molecules involved in the metabolism of gluconeogenesis, glycogenolysis, glycogen synthesis, glycolysis, and other pathways are detected as widely demonstrated in the literature [27,28]. The literature, however, does not present papers that concern the variation of blood parameters in cases and control groups stratified by age classes, and the molecular mechanisms are still unclear.

Instead, aging is a great social and economic challenge that will constantly increase in the coming decades [29], affecting the global population. The liver is one of the main organs that regulates the homeostasis of the body and eliminates toxins. It is well-documented that steatosis is an age-related disease. It is recognized that older people develop the first stage of this disease, which in turn creates a risk for further development of NASH and HCC [30]. In this article, we compared the different food intakes and foods predictive of steatosis in the adult and elderly groups and the different associations between food intake and steatosis. There is little literature comparing food intake between individuals of different ages and in those who develop steatosis. 

Several studies have shown that the dietary pattern of the elderly is influenced by numerous factors, such as socioeconomic factors, food prices, marital status, psychological factors, sensory impairment functioning, access to food, nutritional knowledge and cooking skills, gastrointestinal problems, oral health, and pharmacological factors [31]. 

The changes that accompany aging can influence food choices and eating habits. Nutrition is critical in its contribution to the health of older people and the likelihood of active and healthy aging [32,33,34]. 

Furthermore, differences in food choices between men and women were widely demonstrated in the literature. Women’s higher intakes of fruit, vegetables, and dietary fiber and lower intakes of fat were seen, and women reported a negative perception of the healthiness of sugar, gluten, dairy, red meat, white flour, alcohol, and food additives [35]. In accordance with such healthier food choices, women usually attached greater importance to health [36]. As in this study, the effects of oil intake have been shown to be associated with the development of steatosis, probably due to the intake in terms of calories, and positive effects due to the presence of polyphenols, such as oleuropein, hydroxytyrosol, tyrosol, and caffeic acid, which have important antioxidant and anti-inflammatory effects [37], while the consumption of red meat was predictive in the elderly, and the literature describes a higher risk of developing liver disease in subjects who consume more red meat [38]. Furthermore, the literature declares a neutral role of wheat consumption in the development of steatosis without investigating the age of the subjects [39], while with regard to the consumption of dairy products, there are conflicting results. Indeed, Melkin et al. [40] claim a negative role for the development of steatosis, while Lee et al. [41] attribute to dairy products a protective role associated with the risk of incident steatosis in men and women aged >50 years. Similarly, processed meat in adults and seafood/shellfish in elderly patients have been confirmed to be associated with liver disease [42]. 

In light of these results, we cannot say with certainty that certain foods cause steatosis in the age groups considered, but we can generally state that certain foods are certainly more associated with the disease. Furthermore, the cohort considered is particularly interesting at a geographical level, because it reflects the eating habits of a specific area, halfway between the sea and the hinterland, allowing for a varied consumption of foods of various kinds.

## 5. Conclusions

In conclusion, different ages might have a distinct association with the development of steatosis based not only on blood profile but also on the heterogeneity of food intake. The elderly physiologically eat less and differently from young adults, and this could be the basis of the development of the disease. It is not yet clear how molecular mechanisms and biochemical pathways are involved in the development of this disease. Furthermore, certain foods are associated differently in the two age groups, but this could be explained by the physiological aging of liver cells and related DNA error repair mechanisms. The fact remains that the condition of steatosis appears equally distributed between the two age groups, highlighting how changes in lifestyle are interposed with normal physiological decay. Therefore, future work investigating the association between nutrition and steatosis in different age groups and animal models could be of interest to gain a better understanding of the molecular mechanisms in the development of this disease. Furthermore, the development of new machine learning algorithms will allow the creation of new mathematical models useful for the creation of personalized food plans, not only based on age but also on the basis of other anamnesis and clinical characteristics of the patients, in order to focus attention on an improvement in lifestyle and therefore improve the health status of the subjects. 

Furthermore, the addition of nutrients (macro- and micronutrients) could be useful for understanding new molecular pathways and how these molecules can influence the prediction of clinical outcomes.

## Figures and Tables

**Table 1:** Epidemiological and clinical characteristics of adults/elderly patients with and without steatosis. MICOL Cohort (*n* = 1483).

Parameters *	Total Cohort	Adults (*n* = 726)		*p* ^^^	Elderly (*n* = 757)		*p* ^^^	*p* ^†^	*p* ^¥^
		Steatosis			Steatosis				
		No (*n* = 320)	Yes (*n* = 406)		No (*n* = 334)	Yes (*n* = 423)			
Gender (M) (%)	849 (57.25)	140 (43.75)	269 (66.26)	**<0.001 ^Ψ^**	191 (57.19)	249 (58.87)	0.64 ^Ψ^	**0.01 ^α^**	**0.03 ^β^**
Age (yrs)	64.32 ± 11.35	53.48 ± 6.20	55.63 ± 6.32	**<0.0001**	74.55 ± 6.51	72.79 ± 5.93	**0.0002**	**<0.0001**	**<0.0001**
Degree of Education (%)				**0.01 ^Ψ^**			0.31 ^Ψ^	**<0.001 ^α^**	**<0.001 ^β^**
None	428 (30.70)	18 (6.21)	42 (11.54)		172 (52.44)	196 (47.57)			
Elementary School	427 (30.63)	110 (37.93)	156 (42.86)		61 (18.60)	100 (24.27)			
Secondary School	345 (23.75)	122 (42.07)	131 (35.99)		43 (13.11)	49 (11.89)			
High School	100 (7.17)	40 (13.79)	33 (9.07)		14 (4.27)	13 (3.16)			
Short Degree	94 (6.74)	0 (0.00)	2 (0.55)		38 (11.59)	54 (13.11)			
Smoking Habit (Yes) (%)	192 (13.73)	53 (18.34)	69 (18.80)	0.88 ^Ψ^	40 (12.20)	30 (7.25)	**0.02 ^Ψ^**	**0.03 ^α^**	**<0.001 ^β^**
BMI (Kg/m^2^)	28.43 ± 5.59	25.16 ± 3.46	29.73 ± 4.75	**<0.0001**	26.60 ± 4.04	31.10 ± 5.92	**<0.0001**	**<0.0001**	**0.0001**
Diabetes (Yes) (%)	149 (13.01)	6 (3.17)	18 (7.86)	**0.04 ^Ψ^**	39 (12.15)	86 (21.18)	**0.001 ^Ψ^**	**0.01 ^α^**	**<0.001 ^β^**
Hypertension (Yes) (%)	619 (53.97)	44 (23.04)	103 (44.98)	**<0.001 ^Ψ^**	194 (60.44)	278 (68.47)	**0.02 ^Ψ^**	**<0.001 ^α^**	**<0.001 ^β^**
MetS (Yes) (%)	607 (40.93)	32 (10.00)	167 (41.13)	**<0.001 ^Ψ^**	133 (39.82)	275 (65.01)	**<0.001 ^Ψ^**	**<0.001 ^α^**	**<0.001 ^β^**
*Blood Parameters*									
Glucose (mg/dL)	101.61 ± 25.52	90.12 ± 13.15	100.40 ± 22.05	**<0.0001**	100.32 ± 18.74	112.44 ± 34.52	**<0.0001**	**<0.0001**	**<0.0001**
Cholesterol (mg/mL)	191.96 ± 38.34	198.42 ± 36.03	199.36 ± 38.73	0.61	185.59 ± 39.12	184.99 ± 36.99	0.83	**<0.0001**	**<0.0001**
HDL (mg/dL)	49.50 ± 13.06	54.62 ± 13.21	46.48 ± 12.18	**<0.0001**	51.60 ± 13.60	46.91 ± 11.82	**<0.0001**	**0.002**	0.53
LDL (mg/dL)	122.90 ± 47.76	127.70 ± 32.15	128.86 ± 35.51	0.29	116.29 ± 32.25	117.13 ± 72.55	0.42	**0.0001**	**<0.0001**
Triglycerides (mg/dL)	107.24 ± 64.14	84.27 ± 49.31	122.24 ± 74.00	**<0.0001**	88.55 ± 44.51	124.91 ± 67.68	**<0.0001**	0.06	0.18
Insulin (U/L)	9.40 ± 23.01	6.72 ± 11.39	10.59 ± 6.91	**<0.0001**	8.86 ± 45.55	10.70 ± 8.13	**<0.0001**	0.71	0.73
HOMA-IR	2.52 ± 7.43	1.61 ± 3.98	2.72 ± 2.16	**<0.0001**	2.44 ± 14.55	3.09 ± 3.04	**<0.0001**	0.10	**0.05**
RBC (M/mcL)	4.87 ± 0.51	4.92 ± 0.46	5.06 ± 0.42	**<0.0001**	4.71 ± 0.56	4.79 ± 0.54	**0.02**	**<0.0001**	**<0.0001**
Hemoglobin (g/dL)	14.06 ± 1.52	14.17 ± 1.37	14.70 ± 1.39	**<0.0001**	13.54 ± 1.60	13.77 ± 1.45	**0.03**	**<0.0001**	**<0.0001**
HCT (%)	42.73 ± 3.45	42.10 ± 3.37	43.27 ± 3.37	**<0.0001**	42.99 ± 3.07	42.06 ± 4.43	0.41	0.25	0.10
MCV (fL)	85.78 ± 6.35	86.01 ± 6.97	85.66 ± 5.31	**0.01**	86.43 ± 8.33	84.56 ± 10.09	0.80	0.45	0.15
MCH (pg)	29.00 ± 2.43	28.91 ± 2.59	29.15 ± 2.15	0.33	28.69 ± 2.93	28.16 ± 3.58	0.70	0.88	0.37
MCHC (g/dL)	33.80 ± 1.18	33.60 ± 1.13	34.03 ± 1.20	**<0.0001**	33.20 ± 1.03	33.27 ± 0.90	0.86	0.24	**0.0001**
RDW-CV (%)	13.65 ± 1.16	13.61 ± 1.19	13.61 ± 1.07	0.54	14.12 ± 1.26	14.21 ± 1.68	0.86	**0.04**	**0.05**
Platelets (K/mcL)	227.28 ± 59.32	238.82 ± 55.54	237.86 ± 54.78	0.63	217.46 ± 65.95	216.09 ± 57.49	0.94	**<0.0001**	**<0.0001**
WBC (K/mcL)	6.05 ± 1.98	5.82 ± 2.53	6.33 ± 1.94	**<0.0001**	5.84 ± 1.61	6.13 ± 1.77	**0.01**	0.25	0.06
Neutrophils (%)	57.19 ± 8.40	57.20 ± 8.60	57.14 ± 8.29	0.83	57.61 ± 8.67	57.43 ± 8.21	0.88	0.83	0.98
Lymphocytes (%)	32.13 ± 7.97	32.19 ± 8.20	32.14 ± 7.89	0.95	30.69 ± 7.96	32.00 ± 6.98	0.55	0.44	0.86
Eosinophils (%)	2.88 ± 1.82	2.91 ± 1.82	2.86 ± 1.84	0.59	3.37 ± 2.01	2.63 ± 1.52	0.22	0.34	0.65
Monocytes (%)	7.28 ± 1.77	7.14 ± 1.73	7.36 ± 1.78	0.08	7.76 ± 1.43	7.45 ± 2.13	0.16	0.06	0.90
Basophils (%)	0.52 ± 0.30	0.55 ± 0.33	0.50 ± 0.27	0.08	0.57 ± 0.51	0.49 ± 0.19	0.67	0.54	0.58
Neutrophils (10^3^/µL)	3.51 ± 1.38	3.32 ± 1.16	3.66 ± 1.53	**<0.0001**	3.56 ± 0.97	3.56 ± 1.20	0.90	0.29	0.96
Lymphocytes (10^3^/µL)	1.96 ± 1.48	1.90 ± 2.02	2.02 ± 0.95	**<0.0001**	1.86 ± 0.51	1.94 ± 0.65	0.92	0.49	0.43
Monocytes (10^3^/µL)	0.44 ± 0.16	0.41 ± 0.16	0.46 ± 0.16	**<0.0001**	0.47 ± 0.10	0.45 ± 0.16	0.41	**0.01**	0.51
Eosinophils (10^3^/µL)	0.17 ± 0.13	0.16 ± 0.11	0.18 ± 0.14	0.08	0.21 ± 0.14	0.15 ± 0.08	0.31	0.19	0.48
Basophils (10^3^/µL)	0.03 ± 0.02	0.03 ± 0.02	0.03 ± 0.02	0.39	0.04 ± 0.05	0.03 ± 0.01	0.70	0.84	0.99
HbA1c (%)	37.03 ± 7.49	35.20 ± 6.23	38.44 ± 7.85	**<0.0001**	34.80 ± 5.48	38.90 ± 10.82	0.49	0.97	0.23
Fractional Total Bilirubinemia (mg/dL)	0.91 ± 0.35	0.73 ± 0.38	0.72 ± 0.35	0.87	0.63 ± 0.27	0.65 ± 0.34	0.70	0.07	**0.01**
Direct fractional bilirubinemia (mg/dL)	0.16 ± 0.05	0.16 ± 0.04	0.16 ± 0.05	0.48	0.16 ± 0.07	0.15 ± 0.05	0.69	0.59	0.09
Indirect Fractional Bilirubinemia (mg/dL)	0.49 ± 0.28	0.36 ± 0.06	0.38 ± 0.16	0.99	0.48 ± 0.25	0.51 ± 0.31	0.92	0.56	0.50
GOT (U/L)	22.92 ± 17.33	21.12 ± 5.12	23.13 ± 8.39	**<0.0001**	24.78 ± 32.84	22.60 ± 10.51	0.87	0.74	**<0.0001**
SGPT (U/L)	23.72 ± 16.84	20.90 ± 8.36	26.81 ± 12.89	**<0.0001**	22.41 ± 26.77	23.92 ± 14.18	**<0.0001**	**0.002**	**<0.0001**
GGT (U/I)	21.26 ± 20.19	17.71 ± 12.90	24.08 ± 24.41	**<0.0001**	19.54 ± 17.51	22.58 ± 21.69	**0.0003**	0.42	**0.001**
Albumin (%)	4.14 ± 0.26	4.14 ± 0.26	4.16 ± 0.27	0.53	4.09 ± 0.18	4.04 ± 0.27	0.70	0.46	0.07
Iron (mg/dL)	89.39 ± 30.54	89.87 ± 30.91	89.63 ± 30.83	0.86	77.47 ± 24.08	87.27 ± 25.37	0.21	0.20	0.97
Urea (mg/dL)	40.65 ± 15.77	37.13 ± 9.52	39.17 ± 9.00	**0.03**	42.90 ± 12.68	42.94 ± 24.07	0.31	**<0.0001**	**0.001**
Creatinine (mg/dL)	0.82 ± 0.30	0.77 ± 0.41	0.81 ± 0.17	**<0.0001**	0.96 ± 0.36	0.87 ± 0.21	0.15	**<0.0001**	**0.01**
eGFR (mL/min)	84.78 ± 9.94	86.16 ± 9.28	84.55 ± 9.54	**0.04**	77.25 ± 15.99	81.35 ± 11.16	0.45	**0.001**	**0.01**
AAT (mg/dL)	183.50 ± 39.49	184.34 ± 39.55	184.17 ± 40.56	0.81	165.87 ± 33.47	174.97 ± 22.11	0.73	0.17	0.21
Folate (ng/mL)	8.49 ± 4.92	7.84 ± 3.74	7.34 ± 3.68	**0.02**	9.33 ± 5.65	9.43 ± 5.75	0.47	**0.0005**	**<0.0001**
Vitamin B12 (pg/mL)	363.85 ± 507.63	310.22 ± 139.38	331.03 ± 142.62	**0.006**	430.96 ± 734.27	382.69 ± 661.43	0.59	0.27	**0.03**
TSH (mUI/mL)	889.36 ± 141.83	1819.00 ± 1618.22	1670.66 ± 1542.16	0.17	72.63 ± 500.01	104.16 ± 514.36	0.26	**<0.0001**	**<0.0001**
FT3 (pg/mL)	3.31 ± 0.46	3.45 ± 0.42	3.53 ± 0.38	**0.001**	3.12 ± 0.43	3.15 ± 0.47	0.16	**<0.0001**	**<0.0001**
FT4 (ng/mL)	0.87 ± 0.28	0.83 ± 0.14	0.84 ± 0.14	0.45	0.89 ± 0.160	0.90 ± 0.47	0.13	**<0.0001**	**0.001**
CRP (mg/L)	0.26 ± 0.50	0.18 ± 0.26	0.32 ± 0.60	**<0.0001**	0.12 ± 0.05	0.37 ± 0.73	**0.05**	0.75	0.91

* As mean and standard deviation for continuous variables, and as frequency and percentage (%) for categorical variables. ^^^ Wilcoxon rank-sum test (Mann–Whitney), ^Ψ^ chi-square test, or Fisher’s test, where necessary. ^†^ Wilcoxon rank-sum test (Mann–Whitney) between adults and the elderly without steatosis; ^¥^ Wilcoxon rank-sum test (Mann–Whitney) between adults and the elderly with steatosis; ^α^ chi-square or Fisher’s test, where necessary, between adults and the elderly without steatosis; ^β^ chi-square or Fisher’s test, where necessary, in adults and the elderly with steatosis. Abbreviations: BMI, body mass index; MeS, metabolic syndrome; HDL, high-density lipoprotein; LDL, low-density lipoprotein; HOMA-IR, homeostasis model assessment-estimated insulin resistance; RBC, red blood cell; HCT, hematocrit (he-MAT-uh-krit); MCV, mean corpuscular volume; MCH, mean corpuscular hemoglobin; MCHC, mean corpuscular hemoglobin concentration; RDW-CV, red cell distribution width; WBC, white blood cells; HbA1c, hemoglobin A1c; GOT, aspartate aminotransferase; SGPT, serum glutamic pyruvic transaminase; GGT, gamma-glutamyl transferase; eGFR, estimated glomerular filtration rate; AAT, alpha-1-antitrypsin; TSH, thyroid stimulating hormone; FT3, triiodothyronine free; FT4, thyroxine; CRP, C-reactive protein.

**Table 2:** Dietary daily intake of 28 food groups between age groups, among steatosis and non-steatosis subjects in the MICOL cohort.

Food-Groups *	Total Cohort	Adults (*n* = 726)		*p* ^^^	Elderly (*n* = 757)		*p* ^^^	*p* ^†^	*p* ^¥^
		Steatosis			Steatosis				
		No (*n* = 320)	Yes (*n* = 406)		No (*n* = 334)	Yes (*n* = 423)			
Dairy	74.05 ± 98.95	69.62 ± 90.45	77.55 ± 105.09	0.17	48.09 ± 71.53	65.50 ± 103.08	**0.01**	**<0.0001**	**<0.0001**
Low Fat Dairy	68.20 ± 101.54	64.93 ± 97.19	70.78 ± 104.83	0.30	56.00 ± 90.26	64.64 ± 101.88	0.14	**<0.0001**	**<0.0001**
Eggs	9.02 ± 7.93	9.28 ± 8.39	8.81 ± 7.54	0.33	8.01 ± 6.54	8.26 ± 6.44	0.76	0.11	0.78
White Meat	21.53 ± 28.30	21.10 ± 27.05	21.87 ± 29.26	0.78	14.57 ± 21.79	17.48 ± 29.62	0.10	**<0.0001**	**<0.0001**
Red Meat	25.30 ± 29.28	24.08 ± 31.61	26.26 ± 27.28	**0.01**	17.03 ± 29.94	21.76 ± 28.60	**0.004**	**<0.0001**	**<0.0001**
Processed Meat	4.87 ± 8.61	4.53 ± 9.55	5.13 ± 7.78	**0.01**	3.07 ± 10.48	3.81 ± 6.78	**0.004**	**<0.0001**	**<0.0001**
Fish	20.12 ± 24.31	19.43 ± 24.60	20.66 ± 24.08	0.11	13.95 ± 23.53	17.33 ± 23.29	**0.01**	**<0.0001**	**<0.0001**
Seafood/Shellfish	4.71 ± 10.40	4.37 ± 11.74	4.98 ± 9.21	**0.003**	2.67 ± 5.28	3.88 ± 6.76	**0.001**	**<0.0001**	**<0.0001**
Leafy Vegetables	45.57 ± 61.99	46.28 ± 62.43	45.01 ± 61.68	0.69	32.81 ± 53.35	40.37 ± 58.46	**0.02**	**<0.0001**	**<0.0001**
Fruiting Vegetables	72.44 ± 84.10	70.54 ± 84.97	73.95 ± 83.42	0.30	49.32 ± 71.58	64.78 ± 78.87	**0.02**	**<0.0001**	**<0.0001**
Root Vegetables	14.59 ± 27.31	15.64 ± 28.05	13.77 ± 26.71	**0.01**	11.68 ± 12.63	12.36 ± 22.86	0.28	0.08	0.63
Other Vegetables	66.31 ± 87.96	66.37 ± 87.89	66.26 ± 88.07	0.89	45.58 ± 69.32	58.15 ± 79.62	**0.01**	**<0.0001**	**0.0003**
Legumes	26.40 ± 29.43	25.96 ± 30.34	26.75 ± 28.70	0.84	19.64 ± 22.01	26.19 ± 31.40	**0.01**	**<0.0001**	**0.02**
Potatoes	13.250 ± 16.78	13.12 ± 16.04	13.26 ± 17.35	0.76	12.34 ± 9.71	14.81 ± 21.16	0.78	**0.05**	**0.0005**
Fruits	360.64 ± 447.71	353.08 ± 448.85	366.60 ± 446.99	0.36	282.37 ± 416.41	330.17 ± 424.41	**0.02**	**<0.0001**	**0.0005**
Nuts	3.36 ± 5.91	3.78 ± 6.41	3.04 ± 5.47	**0.005**	2.44 ± 2.92	2.50 ± 4.54	0.30	**0.001**	0.14
Grains	116.08 ± 121.37	111.95 ± 118.24	119.33 ± 123.76	0.30	100.94 ± 124.62	107.44 ± 119.96	0.20	**<0.0001**	**0.0004**
Olives and Vegetable Oil	33.40 ± 37.23	31.62 ± 33.36	34.80 ± 39.99	0.07	29.97 ± 40.38	37.52 ± 51.68	**0.01**	**0.001**	0.59
Sweets	18.79 ± 35.36	19.67 ± 39.75	18.10 ± 31.49	0.83	11.43 ± 18.95	14.92 ± 36.26	0.08	**<0.0001**	**<0.0001**
Sugary	12.71 ± 18.57	13.62 ± 19.58	12.00 ± 17.71	0.21	7.46 ± 11.03	8.90 ± 15.11	0.12	**<0.0001**	**<0.0001**
Juices	9.25 ± 22.46	9.80 ± 24.80	8.82 ± 20.44	0.47	7.59 ± 14.92	7.15 ± 11.38	0.86	**0.01**	0.16
Caloric Drinks	11.35 ± 40.90	9.44 ± 27.72	12.86 ± 47.82	0.06	7.85 ± 17.98	10.92 ± 44.51	0.91	**0.03**	0.56
Ready to Eat Dishes	34.63 ± 45.13	35.37 ± 51.63	34.05 ± 39.26	0.23	17.70 ± 32.64	22.14 ± 35.60	**0.01**	**<0.0001**	**<0.0001**
Coffee	44.71 ± 41.61	43.33 ± 40.25	45.79 ± 42.64	0.33	27.41 ± 32.36	32.46 ± 34.88	0.04	**<0.0001**	**<0.0001**
Wine	109.13 ± 140.77	96.11 ± 123.20	119.41 ± 152.50	0.06	118.27 ± 117.26	128.29 ± 142.47	0.59	**<0.0001**	**<0.0001**
Beer	30.03 ± 79.08	22.18 ± 57.27	36.23 ± 92.29	0.08	20.13 ± 43.72	24.63 ± 67.56	0.29	**<0.0001**	0.17
Spirits	1.66 ± 4.58	1.45 ± 4.19	1.83 ± 4.87	0.21	1.40 ± 3.88	1.58 ± 3.96	0.90	**<0.0001**	**0.01**
Water	666.74 ± 270.02	675.32 ± 277.50	659.97 ± 263.93	0.16	657.08 ± 233.96	644.66 ± 224.52	0.08	0.26	0.87
Total Kcal (die)	2035.34 ± 778.18	1979.34 ± 720.40	2061.70 ± 768.83	0.24	1954.00 ± 747.95	2118.41 ± 858.95	**0.04**	0.58	0.56

* As mean and standard deviation (M ± SD). Food groups were calculated on the quantity of daily consumption (grams). ^^^ Wilcoxon rank-sum test (Mann–Whitney); ^†^ Wilcoxon rank-sum test (Mann–Whitney) between adults and the elderly without steatosis; ^¥^ Wilcoxon rank-sum test (Mann–Whitney) between adults and the elderly with steatosis. Abbreviations: Kcal, kilocalorie.

## Data Availability

The original contributions presented in the study are included in the article. Further inquiries can be directed to the corresponding author.

## Supplementary Materials

The following supporting information can be downloaded at: https://www.mdpi.com/article/10.3390/nu15184058/s1, Table S1: Dietary daily intake of 28 food groups between age groups, among steatosis and non-steatosis subjects in the MICOL cohort, stratified by gender.
