# Supplementary material for: Age-Related Dietary Habits and Blood Biochemical Parameters in Patients with and without Steatosis—MICOL Cohort
Source: Nutrients. 2023 Sep 19;15(18):4058. doi: 10.3390/nu15184058 (PMC10534690; doi:10.3390/nu15184058)
Supplement: Supplementary file 1 [file nutrients-15-04058-s001.zip › nutrients-2573497-supplementary.pdf]

Supplementary Materials

Table S1. Dietary daily intake of 28 food groups between age groups, among steatosis and non steatosis subjects in the MICOL cohort, stratified by gender.

Food-Groups *	Adults (*n=726*)						Elderly (*n=757*)					
	Steatosis						Steatosis					
	No (*n=320*)		p ^	Yes (*n=406*)		p ^	No (*n=334*)		p ^	Yes (*n=423*)		p ^
	Males (*n=140*)	Females (*n=180*)		Males (*n=269*)	Females (*n=137*)		Males (*n=191*)	Females (*n=143*)		Males (*n=249*)	Females (*n=174*)	
Dairy	93.83±100.28	90.75±103.67	0.39	85.11±88.51	99.88±133.27	0.37	44.87±67.25	52.39±76.89	0.75	64.60±108.39	66.79±95.27	0.38
Low Fat Dairy	68.52±106.69	78.70±100.56	0.53	69.84±100.47	91.59±119.39	**0.006**	51.68±84.51	61.78±97.41	0.61	61.80±106.16	68.71±95.59	0.07
Eggs	9.27±7.97	11.64±10.91	**0.03**	9.30±8.85	9.58±7.85	0.28	8.16±7.13	7.81±5.68	0.60	8.70±6.47	7.62±6.35	**0.03**
White Meat	26.88±26.01	28.73±33.10	0.86	24.46±27.00	30.32±30.13	**0.01**	15.20±22.69	13.73±20.59	0.68	15.85±22.55	19.81±37.45	0.31
Red Meat	36.41±30.18	27.56±32.36	**<0.0001**	32.28±26.87	28.29±20.80	0.35	19.22±35.70	14.11±19.56	0.18	24.06±32.64	18.48±21.20	0.34
Processed Meat	6.59±6.71	5.65±9.20	**0.01**	6.97±9.38	5.58±6.33	0.27	3.86±13.44	2.01±3.72	0.06	4.11±7.56	3.39±5.45	0.59
Fish	25.25±25.27	25.09±23.80	0.94	23.57±24.23	25.21±24.85	0.37	14.53±25.55	13.17±20.59	0.55	17.33±23.31	17.33±23.33	0.84
Seafood/Shellfish	5.50±6.80	6.66±20.09	0.19	6.41±13.05	5.59±5.58	0.53	2.92±5.24	2.34±5.34	**0.05**	4.04±5.99	3.64±7.73	0.39
Leafy Vegetables	48.10±50.98	69.87±77.49	**0.007**	41.46±46.56	66.28±87.94	**0.004**	32.93±58.62	32.64±45.56	0.71	37.71±56.15	44.18±61.59	0.09
Fruiting Vegetables	75.49±69.33	106.08±104.60	**0.007**	71.66±73.83	106.73±104.76	**0.0004**	48.93±67.46	49.82±76.98	0.62	62.61±76.41	67.89±82.37	0.37
Root Vegetables	16.29±36.50	22.46±38.24	**0.003**	14.72±31.21	16.27±28.07	0.52	11.13±11.10	12.42±14.42	0.42	11.11±13.82	14.14±31.55	0.58
Other Vegetables	73.00±82.11	99.79±109.74	**0.04**	62.70±77.54	98.25±120.19	**0.001**	42.21±59.18	50.09±80.91	0.97	57.94±82.76	58.46±75.15	0.37
Legumes	30.17±22.13	34.41±43.79	0.86	26.41±26.88	29.18±22.93	0.10	20.84±22.82	18.03±20.84	0.36	25.53±31.86	27.13±30.81	0.37
Potatoes	13.91±14.54	13.97±24.42	0.09	12.75±13.18	9.47±8.90	**0.006**	12.62±10.91	11.95±7.85	0.90	15.61±24.96	13.68±14.03	0.84
Fruits	354.66±412.29	483.07±504.04	**0.02**	388.24±461.56	436.61±477.18	0.15	276.27±389.98	290.51±450.56	0.81	322.02±429.18	341.83±418.45	0.36
Nuts	3.37±4.41	6.57±10.37	**0.03**	3.56±6.27	3.71±6.25	0.39	2.35±2.83	2.56±3.05	0.41	2.26±2.80	2.83±6.23	0.32
Grains	156.90±123.38	97.42±90.94	**<0.0001**	147.51±138.72	100.73±91.21	**0.008**	118.19±142.62	77.91±90.92	0.06	117.96±132.62	92.40±97.49	0.35
Olives and Vegetable Oil	34.09±24.99	32.76±23.04	0.92	31.43±20.51	33.05±23.87	0.88	30.93±33.18	28.67±48.45	0.22	33.52±33.56	43.23±69.61	0.34

(continue)

Food-Groups *	Adults (*n=726*)						Elderly (*n=757*)					
	Steatosis						Steatosis					
	No (*n=320*)		p ^	Yes (*n=406*)		p ^	No (*n=334*)		p ^	Yes (*n=423*)		p ^
	Males (*n=140*)	Females (*n=180*)		Males (*n=269*)	Females (*n=137*)		Males (*n=191*)	Females (*n=143*)		Males (*n=249*)	Females (*n=174*)	
Sweets	26.75±53.88	29.47±50.78	0.67	21.38±25.38	21.46±24.98	0.66	11.85±19.48	10.87±18.26	0.63	12.24±19.86	18.75±51.15	0.11
Sugary	16.56±14.90	22.76±28.94	0.40	15.52±20.27	14.67±18.16	0.85	7.38±10.85	7.56±11.32	0.82	7.74±11.08	10.56±19.39	0.32
Juices	12.50±26.74	11.80±35.43	**0.01**	9.94±21.70	11.75±34.55	0.31	7.19±14.26	8.12±15.80	0.34	7.32±12.16	6.91±10.20	0.50
Caloric Drinks	16.33±47.66	7.03±19.77	**0.001**	17.61±63.17	9.49±20.72	**0.03**	7.33±9.67	8.55±25.15	0.77	9.03±23.58	13.63±63.42	0.07
Ready to Eat Dish	59.49±63.52	49.38±58.10	0.10	47.51±42.13	44.40±32.38	0.83	18.11±26.24	17.15±39.70	0.12	22.77±41.14	21.25±25.76	0.56
Coffee	65.03±40.39	56.02±41.15	**0.03**	61.70±48.15	55.73±39.67	0.30	29.14±34.89	25.09±28.58	0.46	32.35±36.02	32.62±33.27	0.72
Wine	99.95±136.63	51.99±111.39	**0.0001**	144.42±184.04	42.88±66.95	**<0.0001**	147.07±136.14	79.82±69.58	**<0.0001**	161.20±161.22	81.19±92.00	**<0.0001**
Beer	42.08±87.15	10.50±45.35	**0.004**	64.05±128.50	17.41±53.42	**<0.0001**	23.67±52.32	15.82±28.05	0.66	33.60±84.26	11.79±26.00	**0.002**
Spirits	2.70±6.36	0.58±1.58	**<0.0001**	2.70±6.67	0.87±2.30	**0.0002**	1.75±4.98	0.93±1.32	0.22	1.97±4.84	1.02±2.03	**0.002**
Water	672.70±323.54	711.21±309.66	0.17	688.67±305.28	650.89±285.66	0.13	671.35±261.87	638.01±189.66	0.14	639.93±205.18	651.44±250.07	0.94
Total Kcal (die)	2041.46±765.80	1928.87±679.55	0.24	2150.47±747.30	1897.26±784.01	**0.0002**	2048.46±672.36	1822.68±828.30	**0.004**	2202.18±733.59	2008.81±992.14	**0.0007**

* As Mean and Standard Deviation (M±SD). Food groups were calculated on the quantity of daily consumption (grams).

^ Wilcoxon rank-sum test (Mann-Whitney).
